# Adolescents with idiopathic scoliosis and their parents have a positive attitude towards the Thermobrace monitor: results from a survey

**DOI:** 10.1186/s13013-017-0119-x

**Published:** 2017-04-08

**Authors:** Sabrina Donzelli, Fabio Zaina, Gregorio Martinez, Francesca Di Felice, Alberto Negrini, Stefano Negrini

**Affiliations:** 1 ISICO, Via R Bellarmino 13/1, 20141, Milan, Italy; 2 ISICO, Barcelona, Spain; 3 University of Brescia, Don Gnocchi Foundation, Milan, Italy

**Keywords:** Compliance monitor, Brace, Scoliosis

## Abstract

**Background:**

A temperature monitor is used to objectively measure brace wear time in adolescent idiopathic scoliosis. The reliability of this device have been demonstrated, and some specialists introduced the use of a compliance monitor as a standard of care in everyday clinical practice, as we did since 2010 with the Thermobrace (TB). The attitude towards these objective monitors has never been investigated.

The present study aims to investigate the attitude of parents and patients towards the use of temperature sensors for measuring brace wear compliance.

**Methods:**

Three hundred one consecutive girls and 63 boys and their parents have been interviewed. The inclusion criteria were as follows: brace wear full-time prescription at first visit and at least one visit with download and discussion of TB data.

Usefulness, acceptability, reliability, and feeling related to data download were the investigated domains. Patients were invited by the administrative staff to complete anonymously the questionnaire. The European Commission was informed about the present survey and approved it (ICT-37-2015-1). Descriptive statistic was used to present the results.

**Results:**

Among the 364 invited patients and parents, 336 adhered by completing it (rate of responders was 93.2%). The mean age was 14.65 (SD 2.36), the mean Cobb angle was 34.18 (SD 13.57), and the average brace wear prescription was 21.76 h per day (SD 2.53). We did not ask parents about their age, profession, nor other personal data.

Globally, the interviewed patients and parents showed a very positive attitude towards the TB monitor: the mean rate of parents stating a completely or at least partially positive attitude towards this electronic device was 94.0% while among patients, it was 85.6%.

**Conclusions:**

This is the first study investigating the attitude of parents and patients towards a brace wear compliance monitor. People who experienced this objective monitoring are aware of the advantages related to it and support its usefulness not only for clinicians but also for patients and parents to respect the hours prescribed without any affection on the children and parents or the patient-physician relationship. The present results should encourage the spread of these tools in daily clinical practice.

**Electronic supplementary material:**

The online version of this article (doi:10.1186/s13013-017-0119-x) contains supplementary material, which is available to authorized users.

## Background

Adherence is defined as the degree to which a patient acts in accordance to the prescription of a health care provider. It has unquestionable implications on the effect of a therapy and as a consequence on the results of clinical research [1]. The harder the treatment, the higher the risks for non-adherent patients. Brace wear for scoliosis is a very hard and complex therapy; indeed, in this field, adherence can be very challenging due to the difficulties associated with wearing plastic for a long time and during one of the most critical phases of life: adolescence [2, 3]. Many brace studies in the past disregarded adherence to the prescribed regimen or considered referred adherence obtained from surveys and clinical assessments [4] notwithstanding their already recognized low reliability [5].

**Fig. 1:**
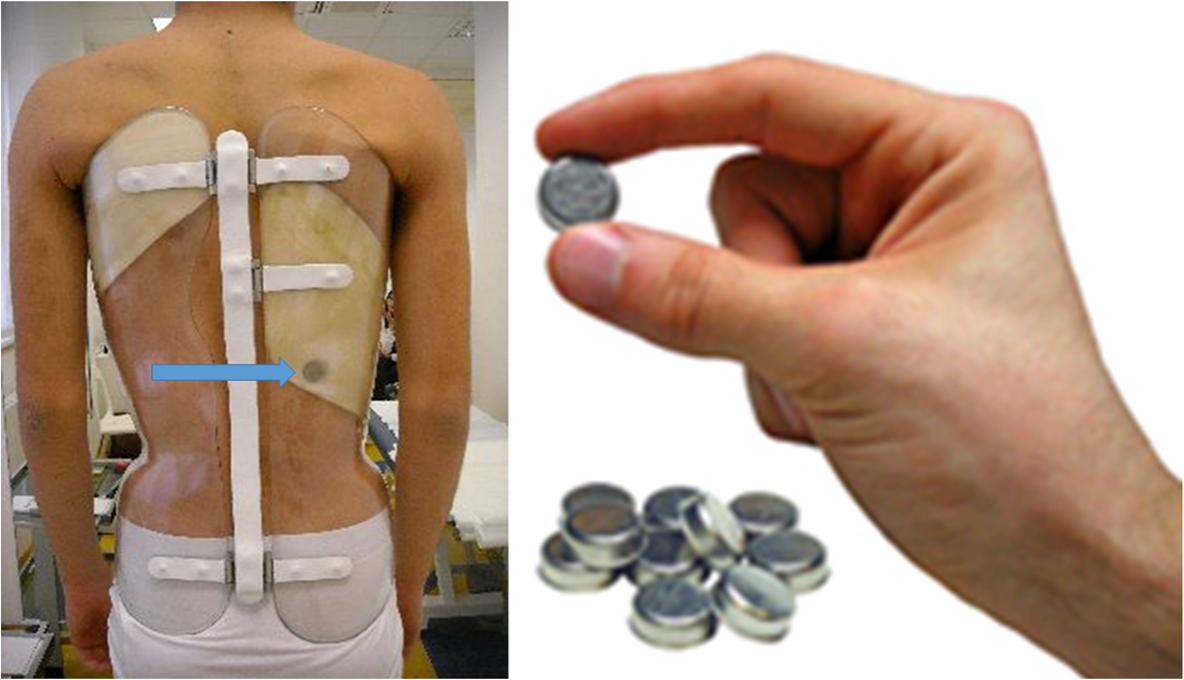
A Thermobrace sensor inside a Sforzesco brace is shown

In recent years, the use of electronic devices was introduced in various research studies; after demonstrating the reliability of temperature sensors, the main topic of the investigation turned to the effects of adherence on results [5–10]. Brace efficacy was recently confirmed by a multicenter RCT [11, 12]; in the same study, adherence was assessed by using objective monitors and a relation between dosage and results was found, thus confirming previous researches: the more the brace wear, the better the results [4, 9–11, 13, 14]. The Thermobrace (TB) is a temperature compliance monitor introduced in everyday clinical activity as a standard of care since 2010, and today, 2579 patients are currently monitored during brace treatment. According to our experience, this device is very helpful in everyday clinical activity, because it offers a valuable help in therapeutic choices, without undermining the relationship with patients and their families [15]. The standardized use of TB contributes by improving the quality of the treatment through an optimization of the dosage, and recently, Karol and colleagues showed that providing patients with feedback about their real dosage increases adherence [16]. Our experience at ISICO is that open discussions based on objective data strengthen the mutual trust needed for the patient-physician relationship and increases compliance. The TB use showed to contribute also to the cognitive behavioral approach needed to enhance compliance [17]. Figure 1 shows the Thermobrace inside a brace.

Even if the usefulness of these electronic monitoring devices in research is widely recognized, in everyday clinical activity, it is still debated with many of the clinicians showing reluctance to its use, despite the clear indication of the SOSORT-SRS consensus [18]. The main reasons they advocate are the fear to harm the patient-physician relationship or to affect the relationship between parents and children being already very fragile at adolescents’ age, but while the trust issue can be sometimes present also among parents who decide not to adopt the Thermobrace, the main reason is probably economic.

The aim of the present study is to investigate, in a population of TB-experienced subjects, the attitude of parents and patients towards the use of temperature sensors for measuring brace wear compliance and to verify the differences between parents and their children in the following domains: understanding of the device, usefulness, acceptance, reliability, and the feeling related to the moment of data reading and discussion.

## Methods

### Participants and procedure

The survey was conducted in a tertiary referral center specialized in scoliosis conservative treatment; the data collection lasted from May 2015 to July 2015. We applied a convenience sampling, inviting all the patients who used a brace and a Thermobrace for at least 4 months. The recruitment was done at the end of each visit; the doctor provided explanations and aims of the survey study and then invited all the participants to fulfill the questionnaire in the waiting room. To allow the maximum rate of responders and considering the sensitive topics treated in the questionnaire, the completion was anonymous. The completed questionnaires were collected by the administrative staff before the patient and the family left our center.

Inclusion criteria were a brace prescription at first visit and at least one visit with download and discussion of TB data, which means at least 4 months of experience with the temperature monitor device and one shared analysis.

All the involved patients were aware of the monitoring device, which was embedded into the brace at the first brace check. Actually, since 2010, the Thermobrace sensor is prescribed to all patients who need a brace to treat their scoliosis. The standardized procedure includes that at the end of the visit, the doctor discusses with the patient and his family the main aim of the treatment and presents the TB as a useful tool able to guide clinical choices and finally to improve the results of the therapy. After these explanations, the parents who accept to buy the Thermobrace automatically agree to accept the monitoring device. At each physiotherapy session (every 3 months) and at each visit (usually every 6 months), the Thermobrace data are analyzed and discussed together to improve brace wear and to understand the main difficulties faced by each patient.

### Thermobrace temperature sensor

To measure the actual brace wear, we used a commercially available device called the “iButton™ DS1922LF5#” (http://www.maxim-ic.com/datasheet/index.mvp/id/4088/t/al) (Maxim Integrated Products, Inc.; 120 San Gabriel Drive, Sunnyvale, CA 94086), which we called for this specific use “Thermobrace.” It is a small temperature data logger to be installed within each orthosis, and it includes a temperature sensor, a battery, and a memory. The sensor is meant to measure the brace temperature, and it is not placed in direct contact with the patient’s skin but within a pressure pad, inside the brace. A reliability study has validated this specific instrument [19].

### Reading software

A specific software program to elaborate the data has been developed and is now freely available online, after a free registration on the website (http://www.scoliosismanager.org/thermobrace). The algorithm used to determine whether a sample corresponds to the brace being worn/not worn, and details concerning the reliability of collected and processed data, has already been described in a previous published paper [15]. This specific software has been integrated with our clinical system; for further details, check also the freely accessible website (www.scoliosismanager.org).

### Survey construction

The conception of the questionnaire was entrusted to SN and SD who have 5 years of experience in using the Thermobrace sensor as a standard of care in braced patients. They decided to use a self-administered questionnaire, mainly composed of multiple-choice questions, to make the completion easier and quicker. Only one question was open, to let the responders share their thoughts freely in respect to what they had understood about the TB sensor. The choice to submit the questionnaire directly at the end of the visit is justified by the optimization of the number of responders; for the same reason, it was agreed that the doctor was responsible for the invitation, as this approach guaranteed a better compliance to questionnaire completion.

The 5 years’ experience with an objective compliance monitor led to the definition of the following main items of the questionnaire:

- *Usefulness and function* of the Thermobrace sensor: the aim was to investigate what the patients and their families understood about these devices.
- *Perceived effect* of the Thermobrace on patients’ behavior (in particular adherence to the treatment), with their brace from both the patients’ and parents’ point of view.
- *Trust affection*: in this item, we aimed to investigate if the awareness of being monitored has any impact on the trust and the patient-parent relationship.
- *Perceived reliability* of the device, which is related to the effective brace wear measured by the device and the declared and perceived mean time of brace wear of patients and parents.
- *Discomfort feelings* related to the moment of data download, reading, and discussion. In these items, all the following feelings are included: awkwardness, anxiety, and being bothered.
- *Satisfaction with the electronic device*: in this item, we aimed to investigate if the participants were happy with the choice of the objective monitoring of brace wear, if they would recommend this use to others, and if they would repeat again their choice.

After a pilot study involving 10 patients, a final version of the questionnaire was defined. Two external investigators, not directly involved in the clinical activity, reviewed the questionnaires and analyzed the data (AN and GM). The decision to make the questionnaire anonymous is justified by the fact that the questionnaire deals with personal and sensitive issues and to avoid the risk that respondents may refuse to answer some questions. It was not possible for parents to see the answers of their children nor vice versa.

### Statistics

For the present study, descriptive statistic was used; in fact, due to the choice of the anonymous completion of the questionnaire, it was not possible to make any kind of correlations with the clinical and demographic data of the included subjects (Additional file 1).

## Results

Among 364 invited patients to complete anonymously the questionnaire, 336 adhered by completing it; therefore, globally, the rate of responders was very high (93.2%), and most of them completed all the required sections of the questionnaire. The mean age of the recruited patients was 14.65 (SD 2.36), the mean Cobb angle was 34.18 (SD 13.57), and average brace wear prescription was 21.76 h per day (SD 2.53). We did not ask parents about their age, profession, nor other personal data.

**Fig. 2:**
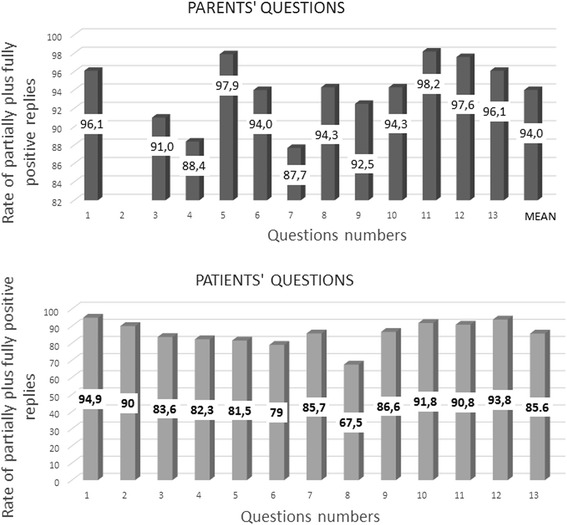
Rates of partially plus fully positive replies obtained from parents’ and patients’ questionnaires (*upper* and *lower parts*, respectively), according to question number and to survey’s items

Globally, the interviewed patients and parents showed a very positive attitude towards the TB monitor; in fact, the mean percentage of parents stating a completely or at least partially positive attitude towards this electronic device was 94.0% while among patients it was 85.6%. The rates of fully positive replies together with the partially positive ones, for each question’s number, for parents and patients are shown in Fig. 2.

The first section of the parents’ questionnaire comprises an open-ended question aimed to investigate the comprehension of the usefulness of the TB sensor. Among the 336 invited parents, seven did not answer to the first question (2.4%). All the answers have been evaluated and classified into three categories: the group of parents who wrote a very detailed description of the TB and its advantages, thus illustrating a very good understanding of the usefulness of the device (8.1%), the group who understood at least partially the usefulness of the TB sensor (88.0%), and those who completely misunderstood the significance and use of the TB sensor (1.5%).

A second question aimed to deepen the usefulness of the TB sensor; in fact, responders had to decide to whom the TB is most useful; multiple choices were allowed. This question showed a rate of responders of 100%. 35.3% of the responders consider the TB useful for parents, 56.9% consider it useful for patients, and 86.5% state that the TB sensor is useful for physicians. Only 0.6% answered “for none” and 1.2% answered “others.” When considering the combined answers, we obtained the following rates: the TB is considered useful for both parents and patients by 22.5% of responders, for physicians and patients by 46.5% of responders, and for physicians and parents by 31.1% of responders. Among those declaring that they do not consider the TB helpful, most of them declare that it is useful for patients and physicians (the percentages are respectively 3.6 and 9.3%). The non-responder rate is 0.8%.

**Fig. 3:**
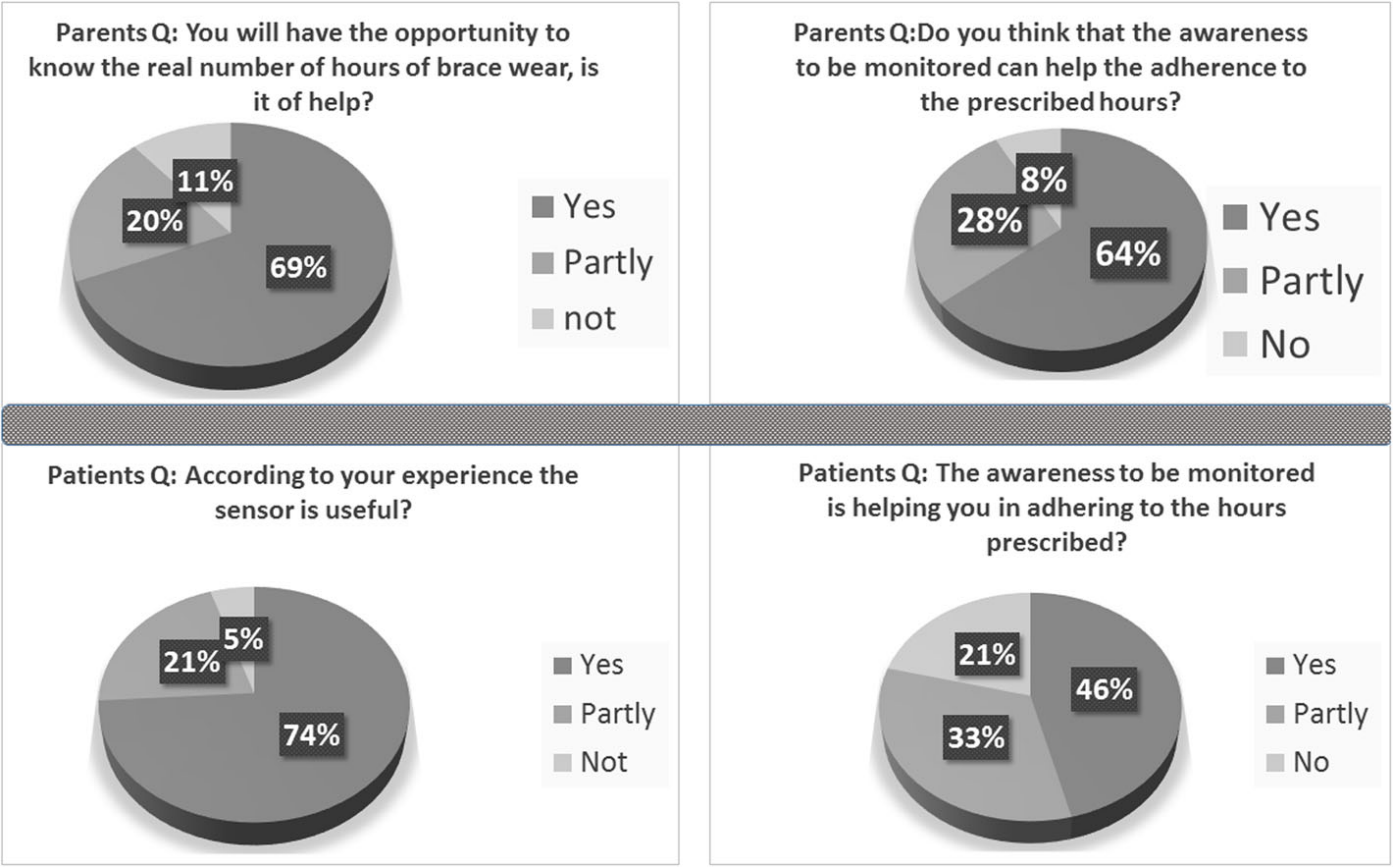
Results from the usefulness item obtained by parents (*upper part*) and patients (*lower part*)

Also, patients were asked about the perceived usefulness; Fig. 3 shows the main results obtained by parents and patients for the usefulness item.

**Fig. 4:**
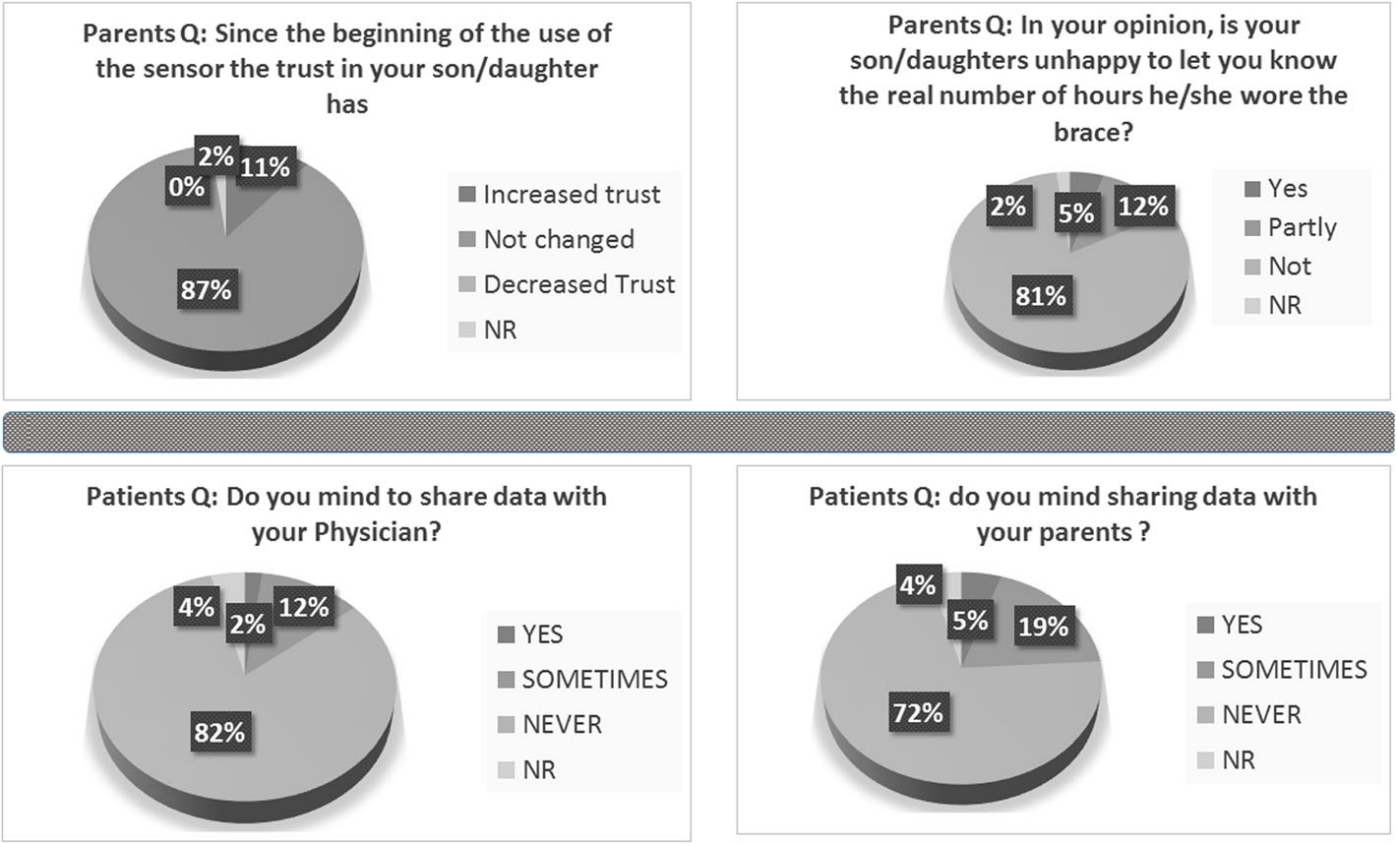
Results from the trust affection item obtained by parents (*upper part*) and patients (*lower part*)

The results concerning the items on trust of parents and patients are shown in Fig. 4.

Table 1 summarizes the rate of answers related to the reliability of the TB sensor and the discomfort feelings related to the moment of data download.

**Table 1:** Summary of the rate of responses related to the reliability of the TB sensor and the discomfort feelings related to the moment of data download of parents (upper part) and patients (lower part) (Additional file 1)

	Question number	Yes	Partly	No	I do not know	NR rate
Items/parents						
TB reliability	7	79.3%	8.4%	0.0%	11.4%	0.9%
Awkwardness	8	4.8%	–	94.3%	–	0.9%
Anxiety	9	6.9%	–	92.5%	–	0.6%
Surprise	10	0.051	–	0.943	–	0.006
Being bothered	11	1.8%		98.2%		0.0%
Items/patients						
TB reliability	5	69.9%	11.6	0.9%	17.6%	0.0%
TB = SPY	3	16.1%	35.9%	47.7%	–	0.3%
TB = ALLY	4	40.1%	42.2%	16.7%	–	1.0%
Awkwardness	7	14.3%	–	85.7%	–	0.0%
Anxiety	8	32.5%	–	67.5%	–	0.0%
Surprise	10	12.8%	–	86.6%	–	0.60%
Bothering	11	8.2%		91.8%		0.0%

*NR* non-responder

In questions 12 and 13, the parents were asked respectively if they would accept again to use the TB sensor and if they would recommend its use to other people. 91.2% answered that they surely would accept again the TB, 6.0% would have some doubts, and 1.5% answered “no” (non-responder rate 0.9%). 91.3% of parents would recommend TB use to other patients, 2.7% declare that they would not recommend the TB use, and 4.8% would recommend the use of TB only in particular situations, for example, if the patient is not accepting the therapy, in very anxious subjects, and in non-compliant patients and if it was possible to check TB data at home by parents (non-responder rate 1.2%).

## Discussion

This is the first study investigating the attitude of scoliosis patients and their family towards a monitoring device able to measure objectively the number of hours of brace wear. The use of compliance monitors in everyday clinical practice is still highly debated among expert clinicians, with very few promoting their use [16, 20, 21], and most of them ignoring their functioning or advantages or simply refusing their use by advocating reasons related to the trust affection and damages to the mutual relationship between patient and physician.

As physicians, we strongly agree that it is fundamental that the new system does not affect negatively the mutual trust needed for the patient-doctor and patient-parent relationship, but after experiencing its use, it is possible to state that the new device provides objective data for open discussions, thus allowing to increase adherence to treatment but without disturbing or making the patient feel worry. This is confirmed by the answers given by the patients and parents involved in the present survey related to the trust items and also by the lower rates of responders referring to feel some discomfort (awkwardness, anxiety, and being bothered) during TB data download and sharing. Considering this controversial issue, it is noticeable that only 1.5% of the patients always mind to show the TB data to his/her doctor; this result confirms the very small impact on the mutual trust between patients and physicians.

In our setting, some parents refused the TB monitoring by saying that they really trust their sons/daughters and hence do not need to monitor them, and therefore, the trust theme rises again but from another point of view. Despite these issues used to justify objective monitor avoidance, the results of the present survey subverted all these assumptions, as most of the parents after having experienced the TB monitoring state that the TB did not affect the trust in their children, and few of them states that the TB sensor increased their trust. Furthermore, we have to emphasize that according to this point of view, only 4.6% of the interviewed patients declare that they always mind to share the data with their parents.

Other reasons related to the difficulties in the spreading of compliance monitor in clinical practice are the economic issues; in fact, the families have to buy this device, even though it is not very expensive. The cost issue is not only related to the cost itself but it also depends on what kind of expenses the families are used to face for treatment. For example, in Italy, braces are provided entirely by the National Health System; in our setting, a private practice facility, the medical visit and the physiotherapy treatment are paid by the patients, and only the brace is provided by the National Health System; therefore, the TB is an additional expense. When focusing on other settings, the economic issue arises again but it is influenced by different factors, for example, in the USA, the treatment costs, brace included, are covered by insurances, but the cost of the compliance monitors is not included and the patient’s family has to pay it with an additional burden of family expenses. The TB sensor is quite cheap, but if parents do not consider it as an essential element for the final result of the treatment, it becomes too expensive. The setting can make the difference, but also the attitude of the professionals involved in the treating team towards this new technology makes the real difference and influences the choices of the patients’ families. In our setting, all the professionals involved in the treating team strongly believe in the TB usefulness. At the beginning, we also had doubts, but this attitude changed immediately after the experimental period in which the TB sensor was gradually introduced in clinical practice, starting from the patients at their first brace prescription, and involving a second time with the patients already under treatment. The use of the TB monitor showed to be useful for clinical decision-making; in fact, at the end of each follow-up visit, the physician is able to prescribe brace wear regimen, by taking into account the results obtained with the real-time brace wear. Dosage adjustments are related to risk factors for scoliosis progression and to the results already obtained. The physiotherapists check the TB data and together with the patients discuss the encountered difficulties in brace wear, to find together possible solutions and advise them to increase their adherence to the prescribed regimen: all our practitioners are fully convinced that all our patients can comply with full time prescription, and we transfer to them this conviction in all the visits. During brace weaning, it is very important to decrease the dosage gradually to avoid correction loss; therefore, the more we are precise in decreasing the time of brace wear, the best are our final results [15, 17, 22]. Globally, we can state that the TB contributes positively to the final results of the treatment [15]. The attitude towards the TB of the professionals involved in the treating team in our specific setting plays a major role in determining the acceptance of the TB by our patients and their families. In fact, among the patients followed in all our facilities around Italy, in the last year, 822 patients accepted to be monitored by the TB and 89 refused it (9.8%); if we consider only the main center in the North of Italy (Milan), last year, 251 patients accepted the TB and 18 refused (6.7%).

One of the reasons for such a low rate of refusals is within the results of the usefulness items of the survey presented in this research study. Most of the parents understand very well how the TB works and what it is aimed to. This is due to the wide explanations provided by the physicians at the moment of brace prescription and due to the team work: all the members of the treating team spread the same message to the patients and their families: the TB can help them in improving results [23, 24]. The treating physician explains always the importance of an objective monitoring for clinical decision-making, and this justifies the fact that the majority of the interviewed parents consider the TB mainly helpful for physicians.

Most of the responders in both groups of parents and patients consider the knowledge of the real-time brace wear helpful; this corresponds to the professionals’ feeling about those who already experienced the effect of the monitoring of the adherence to brace wear and also corresponds to what is already published in the literature, thus confirming the positive effects on adherence of the objective monitoring combined with counseling and cognitive behavioral approach [16, 17].

The perception of reliability declared by the responders, together with the very high level of satisfaction of the parents after their experience with the TB monitor and the fact that most of them strongly recommend the use of the TB monitor to other patients, corroborates the perception which generated the idea of developing a survey to investigate the attitude of the patients and the family towards the TB temperature adherence monitor.

One of the main limits of the present study is the anonymous completion which increased the rate of responders at a very high level but on the other hand limits the possibility to correlate the clinical data to the results of the survey. In future research, it would be interesting to evaluate if the attitude towards the TB is influenced by the severity of the disease. While considering the very large sample surveyed and the very high rates of responders with a positive attitude and acceptance of the device, it is not possible to hypothesize if there is a significant correlation with clinical data.

## Conclusions

The results from the present innovative survey confirm that the experience with objective monitoring gives satisfaction not only to the professionals involved, in addition to their everyday clinical activity, but also to the patients and their families. Indeed, the TB sensor is widely accepted and perceived as very useful for treatment adherence, without affecting either the patient-parent or the patient-physician relationship. The high percentage of parents recommending the use of TB sensor to others can only endorse these findings.

In the light of the present results, we hope and we expect that a popularization of this technology will be encouraged, considering the various advantages which can be shared by all the professionals involved in the treating team.

## Additional file

Additional file 1: The English translation of the survey. (DOC 28 kb)

## Abbreviations

NR: Non-responders
TB: Thermobrace
