# Supplementary material for: Adolescents with idiopathic scoliosis and their parents have a positive attitude towards the Thermobrace monitor: results from a survey
Source: Scoliosis Spinal Disord. 2017 Apr 8;12:12. doi: 10.1186/s13013-017-0119-x (PMC5385080; doi:10.1186/s13013-017-0119-x)
Supplement: Additional file 1: — The English translation of the survey. (DOC 28 kb) [file 13013_2017_119_MOESM1_ESM.doc]

Appendix

The English translation of the survey.

Parents Questionnaire

Please complete anonimously the following questions to help us to improve the use of temperature sensor to monitor brace wear.

1. Are you able to explain in few words what is the sensor and for what we use it? ____________________________________________________________________________________________________________________________________________________________________________________________________________________________________________________________

2. According to you this sensor is useful for: (Multiple-choice answers allowed)

□ The parents □ the patient □ the doctor □ other : _________________ □ noone

3. Do you think that the awareness to be monitored can help the adherence to the prescribed hours?

□ yes □ partly □ No

4. You will have the opportunity to know the real number of hours of brace wear, is it of help?

□ yes □ partly □ No

5. Since the beginning of the use of the sensor the trust in your son/daughter has:

□ increased □ no change □ decreased

6. In your opinion, is your son/daughters unhappy to let you know the real number of hours he/she wore the brace?

□ yes □ partly □ No

7. According to your experience the data collected by the sensor are reliable?

□ yes □ partly □ No □ I don’t know

8. During data controlling made by the doctor or the technician, do you feel uneasy or awkwardness?

□ yes □ No

9. During data controlling made by the doctor or the technician, do you feel anxious ?

□ Yes □ No

10. During data controlling made by the doctor or the technician, do you feel surprised or astonished ?

□ yes □ No

11. During data controlling made by the doctor or the technician, do you feel bothered ?

□ yes □ No

12. if you could come back should you accept again the use of this sensor?

□ surely yes □ I should have some doubts □ surely not

13. Would you recommend to other the use of this device?

□ yes □ No □ yes, but only in case _____________________________________

Patients questionnaire

Please complete anonimously the following questions to help us to improve theuse of temperature sensor to monitor brace wear.

1. According to your experience the sensor is useful?

□ yes □ partly □ No

2. Do you agree with your parents when they decided to adopt the sensor?

□ yes □ partly □ No

3. do you think that the sensor is a spy?

□ yes □ partly □ No

4. do you think that the sensor is an ally?

□ yes □ partly □ No

5. According to your experience the data collected by the sensor are reliable?

□ yes □ partly □ No □ I don’t know

6. The awareness to be monitored is helping you in adhering to the hours prescribed?

□ yes □ partly □ No

7. During data controlling made by the doctor or the technician, do you feel uneasy or awkwardness ?

□ yes □ No

8. During data controlling made by the doctor or the technician, do you feel anxious ?

□ Yes □ No

9. During data controlling made by the doctor or the technician, do you feel surprised or astonished ?

□ yes □ No

10. During data controlling made by the doctor or the technician, do you feel bother ?

□ yes □ No
